# 3D printed ventricular septal defect patch: a primer for the 2015 Radiological Society of North America (RSNA) hands-on course in 3D printing

**DOI:** 10.1186/s41205-015-0002-4

**Published:** 2015-11-27

**Authors:** Andreas A. Giannopoulos, Leonid Chepelev, Adnan Sheikh, Aili Wang, Wilfred Dang, Ekin Akyuz, Chris Hong, Nicole Wake, Todd Pietila, Philip B. Dydynski, Dimitrios Mitsouras, Frank J. Rybicki

**Affiliations:** 1 Applied Imaging Science Lab, Department of Radiology, Brigham and Women’s Hospital, Boston, MA USA; 2 The Ottawa Hospital Research Institute and the Department of Radiology, University of Ottawa, Ottawa, ON Canada; 3 Biomedical Engineering, Materialise, Plymouth, MI USA; 4 Center for Advanced Imaging Innovation and Research, Bernard and Irene Schwartz Center for Biomedical Imaging, Department of Radiology, New York University Langone Medical Center, New York, NY USA; 5 Kosair Children’s Hospital, Lousiville, KY USA

**Keywords:** 3D Printing, Congenital heart disease, Ventricular septal defect, Segmentation, Computed-aided design, Patch, Radiological Society of North America, Hands-on Course, Medical education, Precision medicine

## Abstract

Hand-held three dimensional models of the human anatomy and pathology, tailored-made protheses, and custom-designed implants can be derived from imaging modalities, most commonly Computed Tomography (CT). However, standard DICOM format images cannot be 3D printed; instead, additional image post-processing is required to transform the anatomy of interest into Standard Tessellation Language (STL) format is needed. This conversion, and the subsequent 3D printing of the STL file, requires a series of steps. Initial post-processing involves the segmentation-demarcation of the desired for 3D printing parts and creating of an initial STL file. Then, Computer Aided Design (CAD) software is used, particularly for wrapping, smoothing and trimming. Devices and implants that can also be 3D printed, can be designed using this software environment. The purpose of this article is to provide a tutorial on 3D Printing with the test case of complex congenital heart disease (CHD). While the infant was born with double outlet right ventricle (DORV), this hands-on guide to be featured at the 2015 annual meeting of the Radiological Society of North America Hands-on Course in 3D Printing focused on the additional finding of a ventricular septal defect (VSD). The process of segmenting the heart chambers and the great vessels will be followed by optimization of the model using CAD software. A virtual patch that accurately matches the patient’s VSD will be designed and both models will be prepared for 3D printing.

## Introduction

3D printing refers to the fabrication of a tangible object from a digital file by a 3D printer. Materials are commonly deposited layer-by-layer and then fused to form the final three dimensional object. Additive Manufacturing (AM), Rapid Prototyping (RP), and Additive Fabrication (AF) are synonyms for 3D printing. According to the most recent classification by American Society of Testing and Materials (ASTM), there are seven major types of 3D printing technology. Although these technologies share similarities, they differ in speed, cost, and resolution of the product. Moreover, a variety of materials can be used to fabricate the model.

A handheld printed model derived from Digital Imaging and Communications in Medicine (DICOM) images represents a natural progression from 3D visualization [1]. DICOM image files cannot be used directly for 3D printing; further steps are necessary to make them readable by 3D printers. The purpose of this hands-on course is to convert a set of DICOM files into a 3D printed model through a series of simple steps. Some of the initial post-processing steps may be familiar to the radiologist, as they share common features with 3D visualization tools that are used for image post-processing tasks such as 3D volume rendering.

Most 3D printed models are derived from DICOM images generated from CT scanners. Images can be reconstructed from isotropic voxels with slice thickness less than or equal to 1.25 mm. For 3D printing, image post-processing has both similarities to and substantial differences from methods used by radiologists for 3D visualization. As in 3D visualization, specific software packages enable segmentation of DICOM images using semi-automated and manual segmentation algorithms, allowing the user to demarcate desired parts. The most commonly used tools are thresholding, region growing, and manual sculpting.

The segmented data are then exported in a file format that can be recognized by 3D printers. In essence, this process is conversion of 2D images to 3D triangular facets that compose a mesh surface. To date, the most widely used format is Standard Tessellation Language (denoted by the file extension “STL”). In most cases, the STL output is not optimized for printing and further refinement is required. This refining step may be unfamiliar even to radiologists versed in 3D visualization; Computer Aided Design (CAD) software is used to perform steps such as “wrapping” and “smoothing” to make the model more homogeneous. A key part of 3D printing is choosing the appropriate hardware technology and material. There are several considerations in choosing which technology to use, such as availability, cost, speed, biocompatibility, and most importantly anticipated usage of the product (e.g., a model for surgical planning versus a custom made implant).

Our ultimate goal is to educate participants about the capabilities of 3D printing and, through this hands-on-exercise, provide an initial working knowledge of how it is performed. This session focuses on image post-processing of DICOM image files generated from a CT scan for 3D printing. Participants will learn to segment simple to moderately complicated structures and prepare them for 3D printing. Using this handout as a guide, we will teach participants to use three software packages, ***Mimics*** and ***3-matic*** (Materialise, Leuven, Belgium) and ***Objet Studio*** (Stratasys Ltd., MN, USA).

***Mimics*** is an image-processing package that interfaces between 2D image data (e.g., CT, MRI) and 3D engineering applications. ***Mimics*** is widely used in academics, hospitals, and industry for 3D printing as well as for anatomical measurements, 3D analysis, Finite Element Analysis, patient-specific implant or device design, and surgical planning or simulation. Within ***Mimics***, users can segment any region of interest that can be seen in the medical data and accurately create a 3D model of patient anatomy. ***3-matic*** is a Computer Aided Design (CAD) package dedicated for use with anatomical data. It can perform common CAD operations directly on triangulated STL files. It can also be used to optimize the triangle mesh so the anatomical models can be used in a finite element package. ***Objet Studio*** is a software platform directly connected to the 3D printer that supports STL files from any 3D CAD application. The software offers simple “click & build” preparation and print tray editing. It provides easy, accurate job estimation and full job control.

**Fig. 1:**
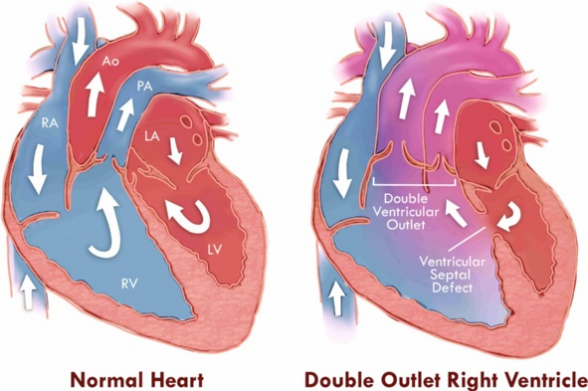
Schematic representation of the anatomy and blood circulation in a normal heart (*left*) and in the case of double outlet right ventricle (*right*)

The patient in this workshop, the 3D Printing Hands-on Course at the 2015 annual meeting of the Radiological Society of North America (RSNA), was born with double outlet right ventricle syndrome (DORV), accompanied by a ventricular septal defect (VSD). DORV is a rare congenital heart disease (CHD) involving approximately 1–1.5 % of all CHD and with an incidence of 3–9 per 100,000 live births [2]. It is defined as the type of ventriculo-arterial connection in which both great vessels (aorta and pulmonary artery) arise entirely from the right ventricle [3]. It is commonly accompanied with a VSD that allows blood to be transferred from the left ventricle to the right ventricle and then to the aorta. DORV is classified based on the location of the VSD in relation to the great vessels: subaortic, subpulmonic, doubly committed, and non-committed [4]. The subpulmonic VSD that is of interest to us in this particular case is a variant of DORV where the pulmonary artery receives oxygenated blood from the left ventricle and into the pulmonary circulation whereas non-oxygenated blood from the RV is streamed to the aorta and thus to the systemic circulation (left to right shunt; Fig. 1).

Treatment requires definite surgical correction and the repair approaches differ on the basis of the subtypes and co-existence of other heart abnormalities. Of importance during repair is the location and the size of the VSD (including the involvement of the conal septum) [4]. For DORV with subpulmonic VSD, the preferred approach is the arterial switch operation along with VSD closure. VSD closure requires a large intra-ventricular baffle/patch sutured into place, closing the ventricular septal defect and redirecting left ventricular outflow to the aorta.

In this course and in order to establish the anatomy of the patient’s heart, we will segment the heart and the great vessels along with the VSD. A custom-made patch, based on the patient’s anatomy and the dimensions of the defect, will be designed. The final output will be 3D-printable STL files of the heart with the defect and the patch. Because time in the Hands-On Session is limited, the RSNA computers have the CT DICOM images pre-loaded, and the software has already been launched. In practice, these simple initial steps require additional understanding of the software.

**Fig. 2:**
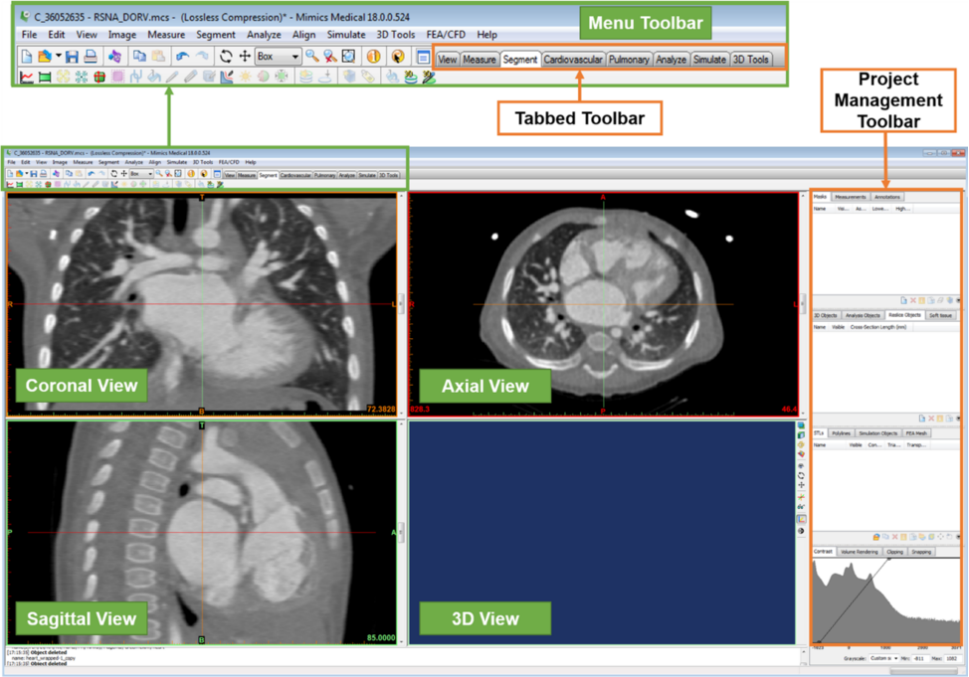
*Mimics* software environment project screen

In summary, we will design a patch for the VSD using contrast-enhanced cardiac CT images, in four tasks: A) segmentation of the heart and the great vessels, B) editing of the heart model in CAD software in order to expose the relevant anatomy, C) designing of a VSD patch and D) preparing the 3D printing of the heart model and the VSD patch. Before we begin with Task A, we will introduce the ***Mimics*** software environment, and more specific the menus, the toolbars and the windows (Fig. 2) as well as useful shortcuts (Table 1).

**Table 1:** *Mimics* software, keyboard and mouse shortcuts

Shortcut	Action
Scroll wheel (mouse center)	Pan: Move the mouse while keeping the scroll wheel pressed
OR Shift right click + drag	
Ctrl + right click + drag	Zoom: Move the mouse vertically while keeping the buttons pressed to zoom in and out
Arrow Up/Scroll wheel up	Go to next slice
Arrow Down/Scroll wheel down	Go to previous slice
Page Up	Skip 10 slices upward
Page Down	Skip 10 slices downward
CTRL + L	Make slice indicators visible/invisible
SPACE	Zoom the chosen view to full screen and back
Backspace	Switch between two window states
CTRL + Z	Undo the previous action.
Right click + drag on images	Adjusts contrast window in images

## Task A: creating a mask of (Segmenting) the heart

### What we are doing

Segmenting the heart. The term “segmentation” describes the task of identifying specific voxels in a region of interest such as the heart chambers and the great vessels. We will isolate the contrast-enhanced chambers and vasculature from the rest of the data in the DICOM images.

### Why we are doing it

To identify the voxels that will eventually be represented in the 3D printed model.

### How to do it

The two segmentation tools that we will be using are “***Thresholding***” and “***Region Growing***”. Both may be familiar from experience with standard 3D visualization. ***Thresholding*** isolates voxels with attenuation within a specified Hounsfield Unit (HU) range. In ***Region Growing***, the user manually identifies a seed point and the software selects voxels within the specified HU range that are physically connected to that seed point.

Each of these steps creates a “**mask**”, namely an intermediate model that could be printed after further manipulations. A list of the masks we will create will appear in the first pane of the Project Management Toolbar (located on the top right of the screen).

1. From the ***Segment*** menu (Fig. 3) in the Menu Toolbar (1), choose  ***Thresholding***. This opens the ***Thresholding*** window where we can specify a HU range. This step creates a mask containing only the pixels that fall within the specified HU range. [*Note: you may also use the* ***Segment*** *tab tools as demonstrated in* Fig. 3 **(1, 2)** *.*] In the **Thresholding** window, set the HU range from 440 to 3071 to eliminate those tissues that fall outside 440-3071 HU **(3)**. Contrast material in the heart and vessels will appear green in the images. Click **Apply (4)**.

**Fig. 3:**
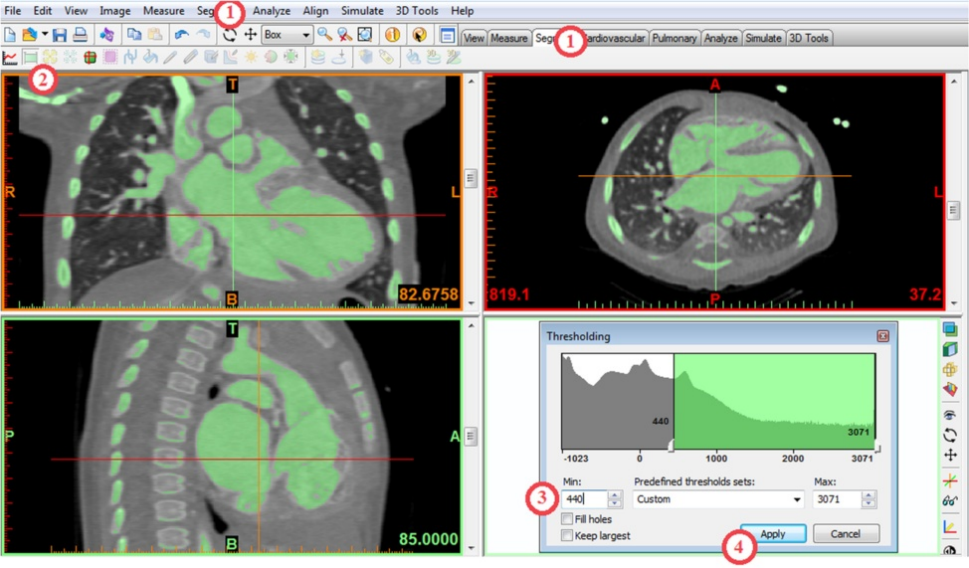
*Thresholding* segmentation process. *Thresholding* can be selected from the Menu Toolbar (1) or from the Segment tab (2). In the Thresholding window (*bottom right*) a HU range from 440 to 3071 is set (3). After clicking Apply (4) the contrast material in the heart and vessels appears green in the images
2. From the ***Segment*** Menu (Fig. 4) **(1)** *,* choose  ***Region Growing*** **(2)**. This tool creates a new mask (yellow) containing only those voxels within the source (green) mask that are connected to the seed point that we identified. This removes all the bones and unrelated structures that fall into the 440–3071 HU range. Left-click on a point **within** the heart in any of the three planes [e.g., click at point **(3)**] to specify a seed point. This will highlight the heart chambers, the large vessels and smaller branches in yellow, while the rest of the highlighted tissue from the previous mask (e.g. bone) remains in green. Close the ***Region Growing*** tool **(4)**.

**Fig. 4:**
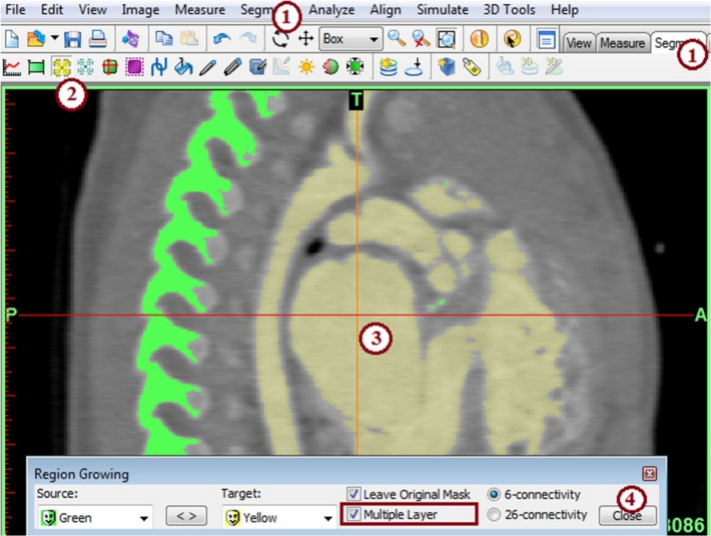
*Region Growing* segmentation process. *Region Growing* can be selected from the Menu Toolbar (1) or from the Segment tab (2). A seed point is selected by left clicking a seed point in the heart atrium (3). Once selected a yellow mask is generated and the Region Growing window can be closed (4)
***Hint: Make sure the***  ***option box is selected. This will perform the operation throughout the entire image stack as opposed to only one cross-section.***
3. Next, we will create a 3D rendering of the heart and vessels from the yellow mask. This intermediate step allows us to visualize the result of the two segmentation steps combined. From the ***Segment*** Menu***,*** choose  ***Calculate 3D***. The *Calculate 3D* window will show up (Fig. 5). Ensure that the yellow mask is highlighted and the **Quality** is set to “Optimal” and hit the **Calculate** button to create a 3D object.

**Fig. 5:**
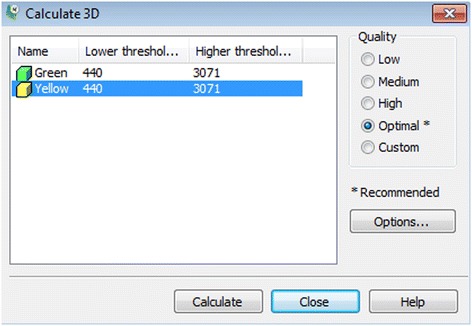
Calculate 3D window. The Yellow mask is selected and Quality is set to optimal
  The 3D object we have created appears in the *3D View* window as well as on the second pane of the **Project Management** toolbar. To adjust the visualization in the *3D View* window, zoom with the mouse wheel and pan by holding the wheel down and moving the mouse. To show the rendering on the full screen, either hover the mouse cursor over or click on the *3D View* window (the bottom right image) and hit the spacebar. For better visualization we can toggle between the CT reference planes by selecting from the ***View*** menu, ***3D Viewports*** and checking or unchecking ***Reference Planes***. The screen can be reset to the 4-image view by hitting the spacebar again. Note that at any point in time, you may hide or show the 3D object by clicking on the **eyeglasses**  in the Project Management Toolbar (Fig. 6) **(1)**.

**Fig. 6:**
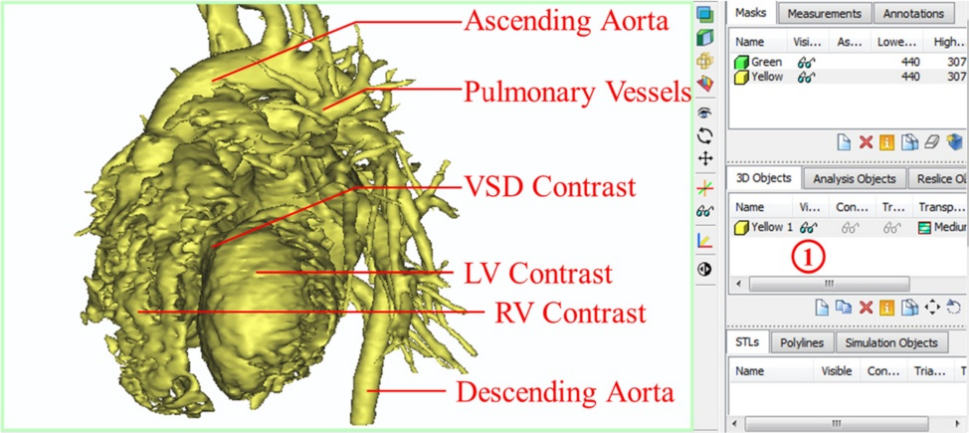
3D View of the created 3D Object –Yellow (1). Review of the anatomical structures
***Note*** *: We have now calculated a 3D surface model of the region that was highlighted in the “Yellow” mask. This model is an STL file that describes the geometry as a set of connected triangles. STL is the file format needed to create a 3D printed geometry. This differs from a volume rendered model (i.e., 3D visualization) in that it contains exportable surfaces.*
4. Finally, we will rename our 3D Object to “**Heart Contrast**” in order to facilitate the following steps. To do this, left-click on the current name of the 3D object (Fig. 7), **Yellow 1** in the 3D Objects list in the Project Management Toolbar **(1)** and type in **Heart Contrast**, then press  on your keyboard.

**Fig. 7:**
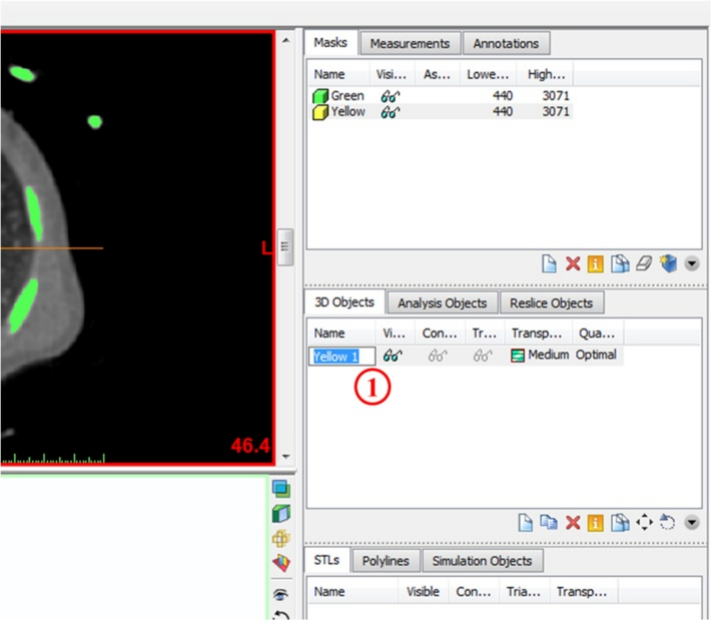
Renaming the 3D Object–Yellow (1) to Heart Contrast

## Task B: exporting the 3D model and editing in 3-matic

### What we are doing

Exporting the STL file from ***Mimics*** to ***3-matic***, which is a CAD software so as to perform a number of post-processing steps. In ***3-matic***, the STL file will undergo two smoothing steps, namely the “***Wrapping*** *”* and an additional level of smoothing called “***Smoothing*** *”*. Then, the heart and vessel wall will be rendered around the heart chambers and vessel lumen. The STL file will finally undergo trimming and the intracardiac structures will be revealed.

### Why we are doing it

To refine our 3D model and to eliminate surface imperfections in the 3D printed model. “***Wrapping*** *”* will fill any holes and create a smooth watertight surface. The extra level of smoothing (“***Smoothing*** *”)* is needed in order to decrease the amount of “noise” that is introduced during the scanning process. We will create a rendering of the heart chambers and the vasculature wall rather than the opacified intraluminal blood pool so that the printed model better represents the septal wall.

### How to do it

We will export the final iteration of our STL file from ***Mimics*** into ***3-matic***. In 3-matic, we will perform the “***Wrapping*** *”* and an additional smoothing step (“***Smoothing*** *”)*. A rendering of the heart and vessel walls will be generated using the *“* ***Hollow*** *”* tool. We will then obtain an intracardiac view of the VSD and the outflows of the great vessels within the hollowed model after trimming the heart into two portions using the “***Trim***” tool.

**Fig. 8:**
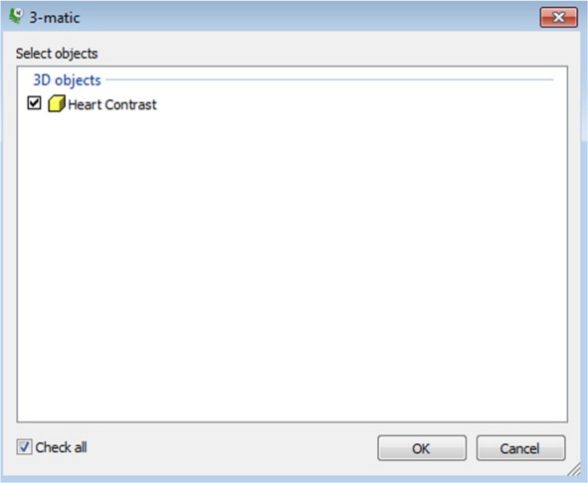
Exporting from *Mimics* to *3-matic*

**Fig. 9:**
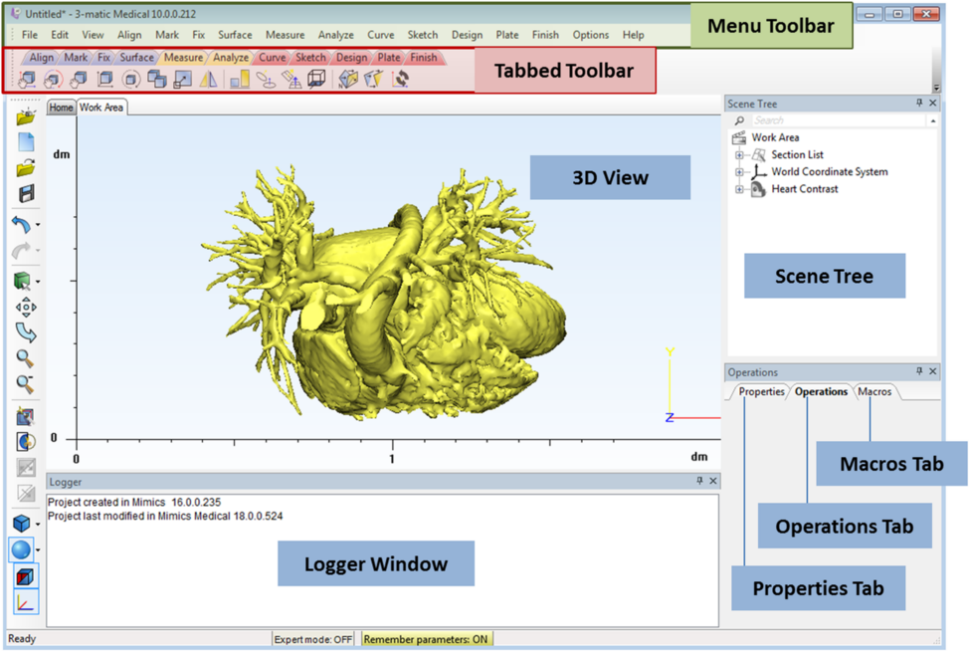
*3-matic* software environment project screen

1. From the ***File*** Menu***,*** select ***Export,*** and then ***3-matic.*** In the dialog box that appears (Fig. 8), select the 3D Object named **Heart Contrast** and click OK to launch the 3-matic software so as to begin the refinement and editing. At this point we will introduce the ***3-matic*** software environment, and more specific the menus, the toolbars and the windows (Fig. 9) as well as useful shortcuts (Table 2).

**Table 2:** *3-matic* software, keyboard and mouse shortcuts

Shortcut	Action
Scroll wheel (center mouse)	Pan: Move the mouse while keeping the scroll wheel pressed
OR Shift + right click + drag	
Ctrl + right click + drag	Zoom: Move the mouse vertically while keeping the buttons pressed to zoom in and out. Alternatively, use the mouse wheel.
OR Scroll Wheel Up/Down	
Right Click + Drag	Rotate model
	*Hint: To rotate the 3D Object, make sure to select it by either left-clicking on it, or by selecting it from the Scene Tree*
Numeric Keypad 8	View from top
Numeric Keypad 6	View from right
2. Apply the ***Wrap*** operation. First, from the **View** menu select Default Views and then left click on Top (*Hint: you can also press the* ***numeric keypad 8 key***). Then, select the ***Heart Contrast*** model from the Scene Tree (Fig. 10 **)**. Now from the **Fix** Menu, choose **Wrap**  to eliminate gaps and smooth rough areas on your model. The ***Gap Closing Distance*** should be set as default at 0 mm. The gap closing distance refers to the largest separation between points for which anatomic variations will be smoothed. The ***Smallest Detail*** in the new window should be set at 0.5 mm; this is on the order of the size of the CT voxel. Rough areas that represent image noise will be eliminated.

**Fig. 10:**
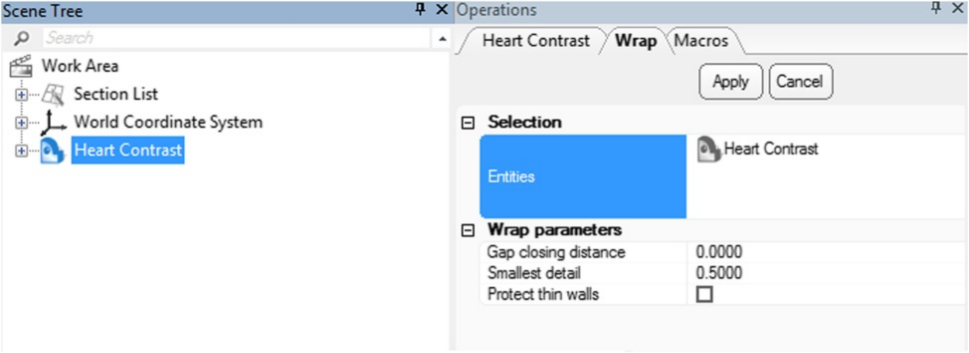
*Wrap* operation. Scene Tree and Operations tab selections
  You obtain a wrapped model, named **Heart Contrast_wrapped**, which appears at the ***Scene Tree***. At this point, we will have two 3D objects overlapping: the **Heart Contrast** model and the **Heart Contrast_wrapped** model. We want to visualize only the latter. On the ***Scene Tree***, right-click on the original 3D model icon **(Heart Contrast)** and click ***Hide*** (Fig. 11).

**Fig. 11:**
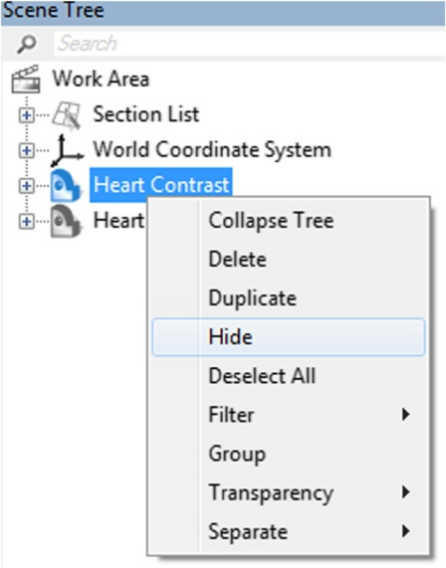
Toggling between the visibility of the original and the wrapped Heart Contrast model
3. Apply the ***Smooth*** operation. First, click the ***Heart Contrast_wrapped*** model icon from the Scene Tree so that the wrapped 3D model appears as the **Entities** value. Then, from the **Fix** Menu, choose  ***Smooth***. Set a ***smooth factor*** of 0.3 and click the **Apply** button (Fig. 12). Of note, this operation will not result in the creation of a new 3D object on the ***Scene Tree***.

**Fig. 12:**
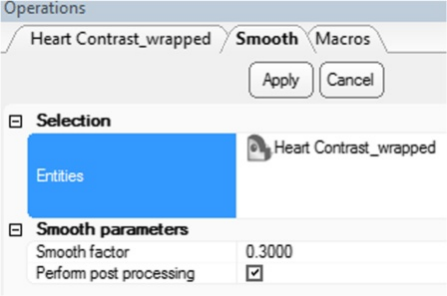
*Smooth* operation. Operations tab selections
4. We will now use the wrapped 3D model that represents the blood pool in order to create an approximation of the inner surface of the walls of the heart and the vasculature. To do this, the contrast in the heart chambers and the vessel lumen will be offset with a constant wall thickness. First, from the **Design** Menu select ***Hollow*** . Click on the ***Heart Contrast_wrapped*** model icon from the ***Scene Tree*** so that the wrapped 3D model appears as the **Entities** value. Set the ***Hollow type*** to outside, the ***Distance*** (which represents the wall thickness) to 1.5 mm and the ***Smallest detail*** (the size of the triangles of the newly created wall) to 0.75, as shown in Fig. 13.

**Fig. 13:**
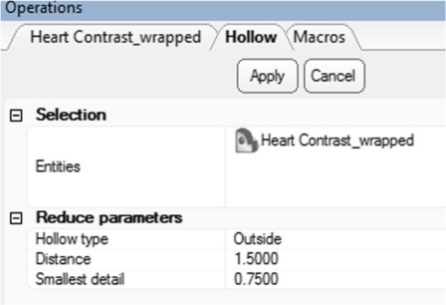
*Hollow* operation. Operations tab selections
  As with the ***Smooth*** operation, this will not result in the creation of a new 3D object on the ***Scene Tree***, rather will “inflate” the current 3D model. Figure 14 shows the model before (left) and after (right) applying the ***Hollow*** operation.

**Fig. 14:**
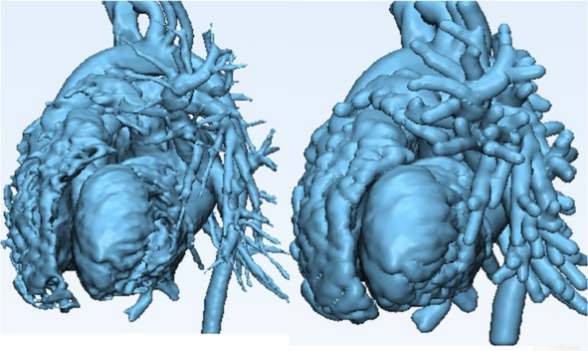
*Hollow* operation end result. Note the differences between the model pre (*left*) and post adding a wall thickness (*right*)
5. In order to visualize the VSD and the outlets of the great vessels, we need to obtain an intracardiac view by cutting open the ventricles. This will be performed, using the ***Trim*** tool. First, we will position the heart for trimming. From the **View** menu select **Default Views** and then left click on **Top** (*Hint: you can also press the* ***numeric keypad 8 key*** **).** This will allow us to view the model from above. From the **Finish** Menu select ***Trim*** . Select the wrapped and hollowed model (***Heart Contrast_wrapped)*** in the ***Scene Tree*** so that it appears as the **Entities** value. Set the parameters as shown in Fig. 15: choose ***Remove outer*** (the area outside the trimming outline will be trimmed away) in trimming method and make sure that ***Fillet*** is unchecked.

**Fig. 15:**
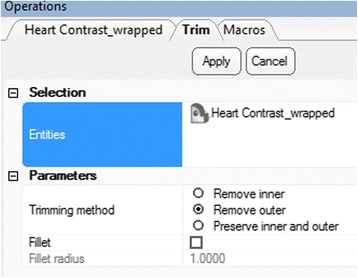
*Trim* operation. Operations tab selections
  We shall now trim away part of the hollowed model so as to expose the ventricular septal defect. This will further allow us to design the patch. To do this we will draw a triangle by indicating three landmark points as described below and visualized in Fig. 16:
  - The first landmark point will be set between the left and the right ventricle.
  - The second landmark point will be set between the anterior aspect of the right atrium and the pulmonary vasculature.
  - The third and final landmark point will be set between the lateral aspect of the left atrium and the pulmonary vasculature. You will then connect the third landmark point with the first point (a), thus designing a triangle.
    Click on **Apply** in the **Operations** tab. The portions of the heart outside the indicated triangle will be removed, while the inside portions will be preserved. Of note, this will not result in the creation of a new 3D object in the ***Scene Tree***.

**Fig. 16:**
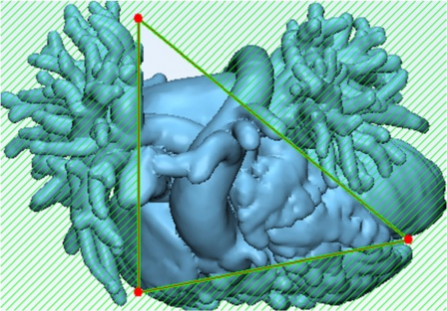
Exposing the ventricular septal defect. Trimming the hollowed heart model by designing a triangle by connecting 3 distinct landmark points

**Fig. 17:**
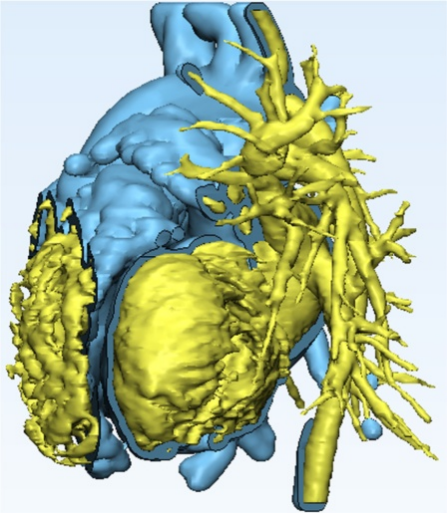
Reviewing the anatomy after revealing the ventricular septal defect. The initial (*Heart Contrast*) model in yellow and the hollowed model (*Heart Contrast_wrapped)* representing the wall of the heart chambers and the vasculature model in blue are superimposed in order to appreciate the anatomical relationships

To review the anatomy, we will orient the trimmed model appropriately by selecting from the **View** menu, **Default Views** and then left clicking on **Right** (*Hint: you can also press the* ***numeric keypad 6 key***). This will show the trimmed heart model from the right aspect. Now, make the initial **Heart Contrast** model visible by right-clicking on **its icon** in the ***Scene Tree*** and selecting **Show**. You will appreciate the relationship between the 3D model that represents the contrast-enhanced blood pool from the initial CT scan (***Heart Contrast*** **)** and the hollow model representing the wall of the heart chambers and the vasculature (***Heart Contrast_wrapped)*** (Fig. 17).

**Fig. 18:**
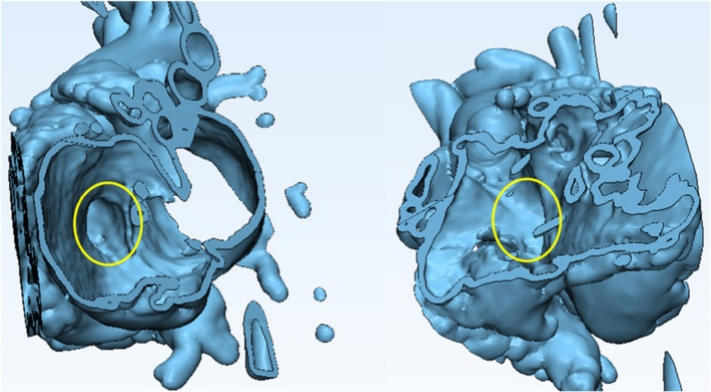
Intracardiac view of the ventricular septal defect (highlighted with a *yellow circle* below) and the outflows of the aorta and the pulmonary artery. *Left panel* shows view from right and *right panel* shows view from left

Once satisfied with the review of the anatomy, hide the initial **Heart Contrast** model by right-clicking on **its icon** and selecting **Hide** on the ***Scene Tree*** menu. You will now be able to view the internal structures of the two ventricles, including the VSD (highlighted with a yellow circle below) and the outflows of the aorta and the pulmonary artery (Fig. 18).

**Fig. 19:**
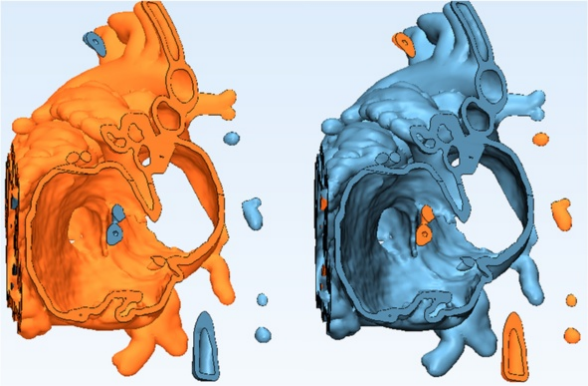
Removal of floating shells. Clicking on a point anywhere on the model in the marked area turns orange (*left*). Applying the *Invert Marking* transfers the marked area from the heart to all floating shells

Following the trimming process we will notice a number of unconnected remnants that are defined as floating shells. These will now be selected and removed. (*Hint: A shell is defined as a limited collection of triangles correctly connected to each other.)* From the **Mark** Menu select ***Shell***  *.* Click on a point anywhere on the ***Heart Contrast_wrapped*** model in the **3D View** screen. The marked area will turn orange and appears in the ***Scene Tree***. From the **Mark** Menu select ***Invert Marking***  *.* This will transfer the marked area from the heart to all floating shells. (Fig. 19). Hit the  key on your keyboard to discard the marked floating shells.

## Task C. Designing a virtual patch for the VSD

### What you are doing

We will design a virtual patch that covers the VSD. The patch will be first manually drawn to accurately match the patient’s anatomy. Then, the corresponding surface will be created and a wall thickness will be added to it.

### Why you are doing it

***3-matic*** provides the ability to design custom devices/patches based on the patient’s anatomy.

### How to do it

We will use the **Create Curve** module to draw the patch on the trimmed 3D model of the heart chambers that gives us the ability to view the VSD from within the heart. This curve will then be transformed into a surface by applying the **Fix Hole** module. A wall thickness will be added to the surface, thus giving it the width of the intraventricular septum, using the **Hollow** operation.

**Fig. 20:**
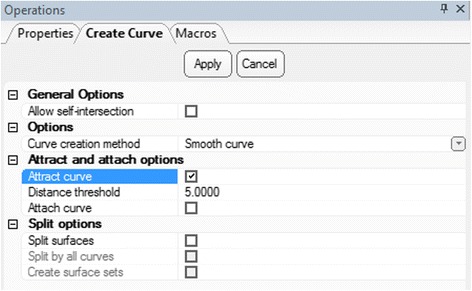
*Create Curve* operation. Operations tab selections

**Fig. 21:**
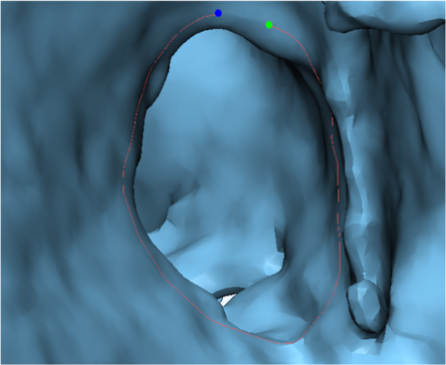
Creating the ventricular septal defect Curve. Note that the curve is not closed

**Fig. 22:**
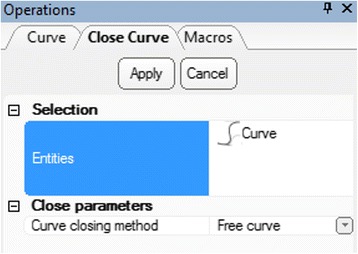
*Close Curve* operation. Operations tab selections

**Fig. 23:**
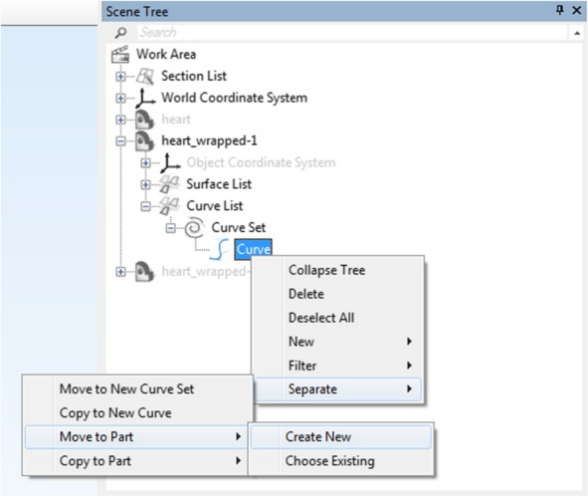
Creating a new ventricular septal defect 3D object that will represent the *Patch*

**Fig. 24:**
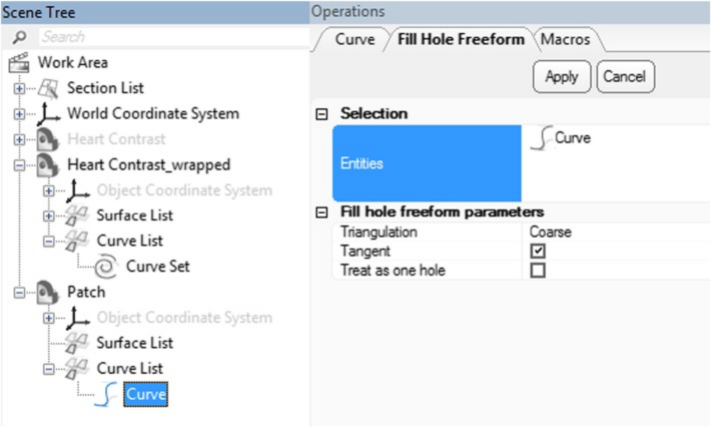
*Fill Hole Freeform* operation. Scene Tree and Operations tab selections

**Fig. 25:**
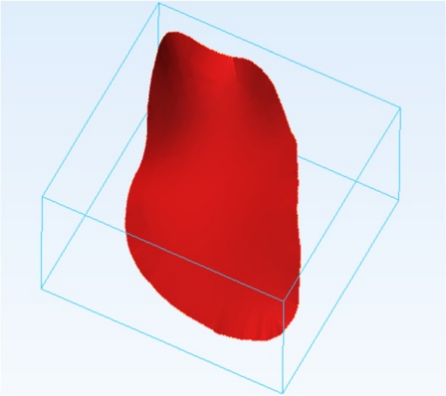
*Fill Hole Freeform* end-result. A ventricular septal defect filling surface (representing the Patch) with no wall thickness

**Fig. 26:**
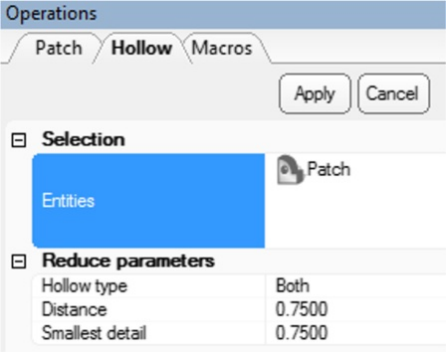
Adding a wall thickness to the *Patch* using the *Hollow* operation. Operations tab selections

**Fig. 27:**
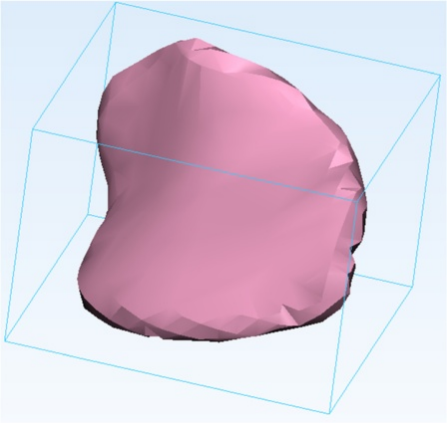
*Hollow* operation end-result. The *Patch* now has obtained the needed wall thickness

**Fig. 28:**
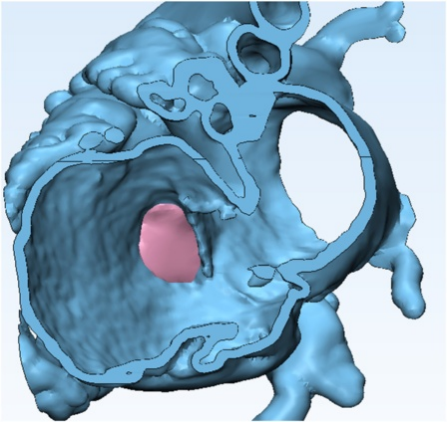
Visualizing the accurate matching of the *Patch* and the ventricular septal defect

1. Select ***Curve*** from the Menu Toolbar and use the ***Create curve***  operation to draw the curve that will be used to create the patch. In the ***Create curve*** tab under **Operations**, set curve creation method as ***smooth curve***, check ***attract curve***, and set distance threshold to ***5.0*** (Fig. 20). Ensure that you rotate and zoom in on the ventricular septal defect. We will use landmark points to indicate the contour of the ventricular defect by left clicking to place each point. We recommend that you begin and end the curve at an area that is easy to visualize. *(Hint: if you feel you have made an error, you may always undo any operation by pressing*  *on your keyboard).* It is important that you do not close the curve, as shown in Fig. 21. Once you are satisfied with your outline of the patch, press  key on your keyboard and then left click on **Cancel** in the **Operations** window. On the ***Scene Tree*** menu a  ***Curve*** item will appear and we will select it by left clicking. Now, return to the **Curve** menu and select the **Close Curve** operation which will connect the first and the last points of this curve. Ensure that the newly created  ***Curve*** is in the entities list and that the curve closing method is **Free Curve** and press Apply (Fig. 22).
2. To create a new VSD patch object, we will right mouse click on the  ***Curve*** in the ***Scene Tree*** and select Separate > Move to part > Create New as depicted in Fig. 23. A new object (***Heart Contrast_wrapped-001***) appears in the ***Scene Tree*** and this will represent the patch. Change name to **Patch** by left mouse clicking on it in the ***Scene Tree***, typing in **Patch** and pressing  on your keyboard. We should hide now the ***Heart Contrast_wrapped*** model and leave only the patch visible.
3. In the next step we will fit a surface onto this curve using the ***Fill Hole Freeform***  operation from the **Fix** menu. Under the **Patch** object in the ***Scene Tree***, select ***Curve*** under ***the Curve List***. In the ***Operations*** tab and under the Fill Hole Freeform parameters, the ***Triangulation*** method should be set to Coarse, the ***Tangent*** should be selected and then click **Apply** (Fig. 24)*.*
  This operation produces a surface that fills the ***Curve*** which we have drawn around the VSD, albeit creating a surface without thickness (Fig. 25).
4. Add thickness to the surfuce. We will use the ***Hollow*** tool as in **Task B**. First, click the ***Patch*** model icon from the ***Scene Tree*** so that the ***Patch*** appears as the **Entities** value. From the **Design** Menu select ***Hollow*** . Set the ***Hollow type*** to both, the ***Distance*** (which represents the wall thickness) to 0.75 mm and the ***Smallest detail*** (the size of the triangles of the newly created wall) to 0.75 mm, as shown in Fig. 26. Click on **Apply** to produce the patch with the wall thickness (Fig. 27).
  Now you may examine how the VSD patch fills the defect by making the rest of the heart outline visible. Right mouse click on the icon of the ***Heart Contrast_wrapped*** object and select **Show**. Rotate, pan, and zoom as necessary to locate the patch inside the heart model (Fig. 28).
5. As a last step we will export the STL files of the trimmed Heart model (***Heart Contrast_wrapped)*** and the VSD patch (***Patch)***. Make sure that both the ***Heart Contrast_wrapped*** model and the ***Patch*** appear as the **Entities** value. Select **File** >**Export** >**Export as binary STL**. Select the **Desktop** as the destination directory and click on **Apply**.

## Task D: 3D printing the heart and the VSD patch STL files

### What you are doing

Using a new software platform called ***Objet Studio*** to place the heart model and the VSD patch on the “build tray”, the platform on which the model will be built. After visualizing the heart and the patch from different perspectives, we will assign the material and “send” the job to the 3D printer.

### Why you are doing it

In ***Mimics*** and ***3-matic***, we have generated the STL file. ***Objet Studio*** is a software package associated with the 3D printer and it is used to organize print jobs, select the materials, and execute printing.

### How to do it

The STL file will be imported into the new software package. Next, the file will be rendered exactly as it will be 3D printed on the build tray. The orientation can then be adjusted, and the material of choice selected. Build time and material usage will also be estimated.

We have now switched software packages and will simulate the printing of the heart and VSD model using ***Objet Studio***.

**Fig. 29:**
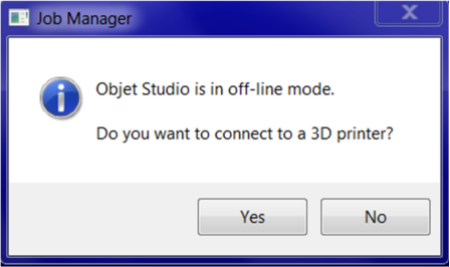
*Objet Studio* Server connection window

**Fig. 30:**
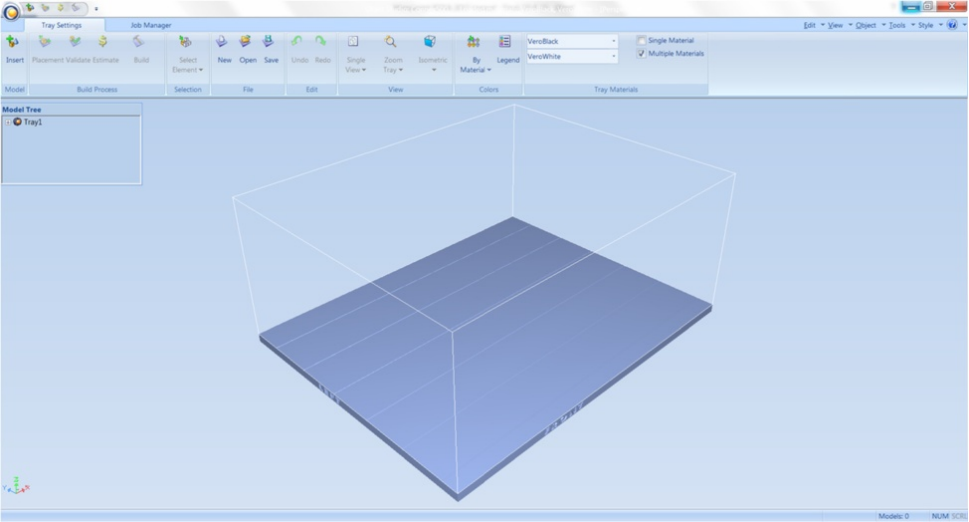
*Objet Studio* software environment project screen

**Fig. 31:**
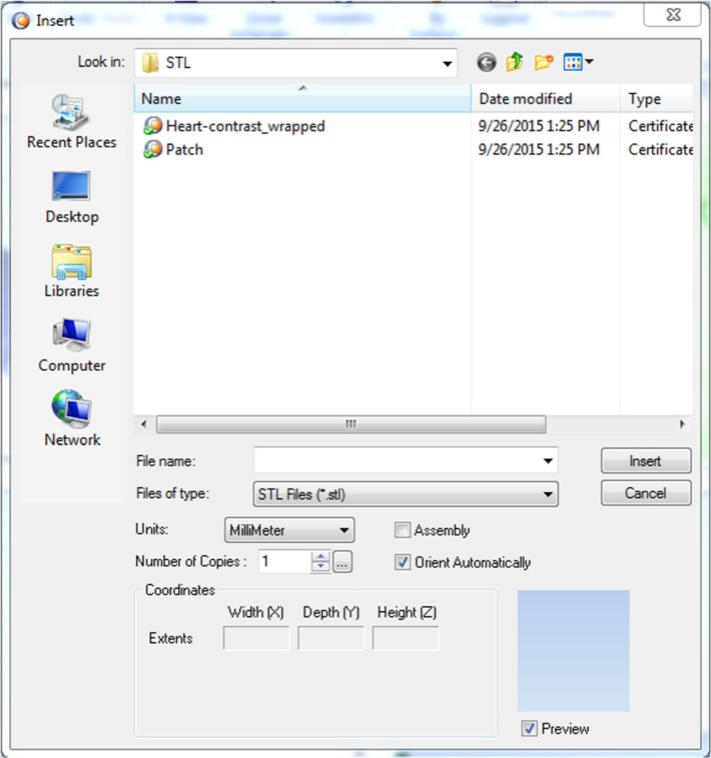
Inserting the STL files of the patch and the trimmed heart models to *Objet Studio*

**Fig. 32:**
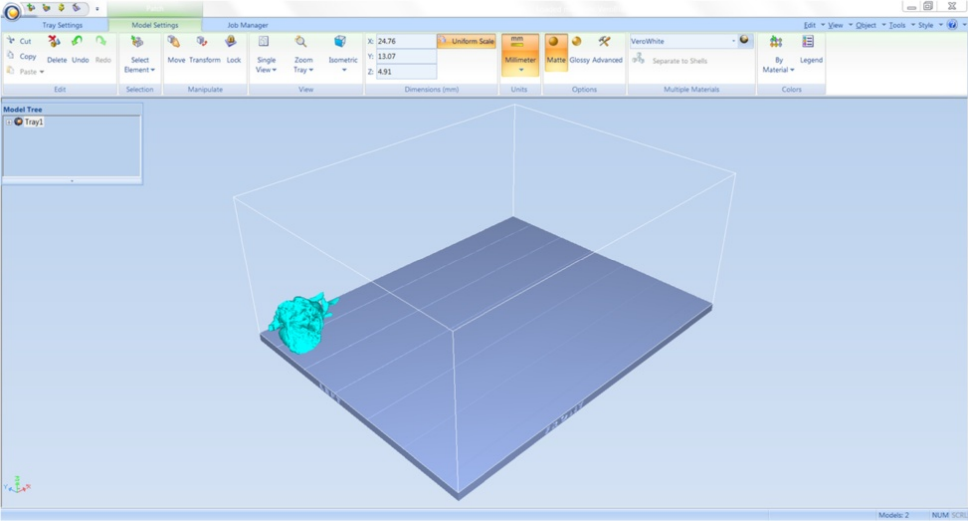
*Objet Studio* software representation of the build tray of the printer with the two models imported and automatically oriented

**Fig. 33:**
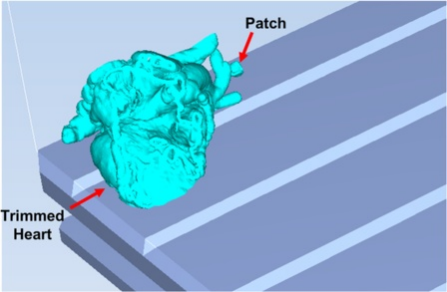
Single zoomed Isometric view of the models on the build tray. The Trimmed Heart and the Patch models are indicated

**Fig. 34:**
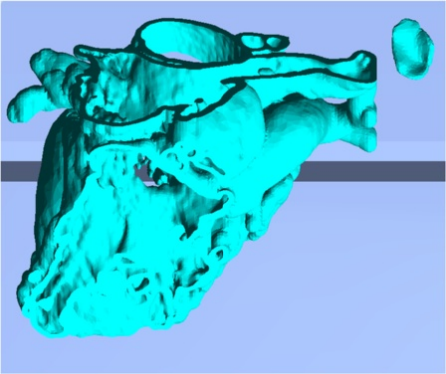
Single zoomed Top view of the models on the build tray

**Fig. 35:**
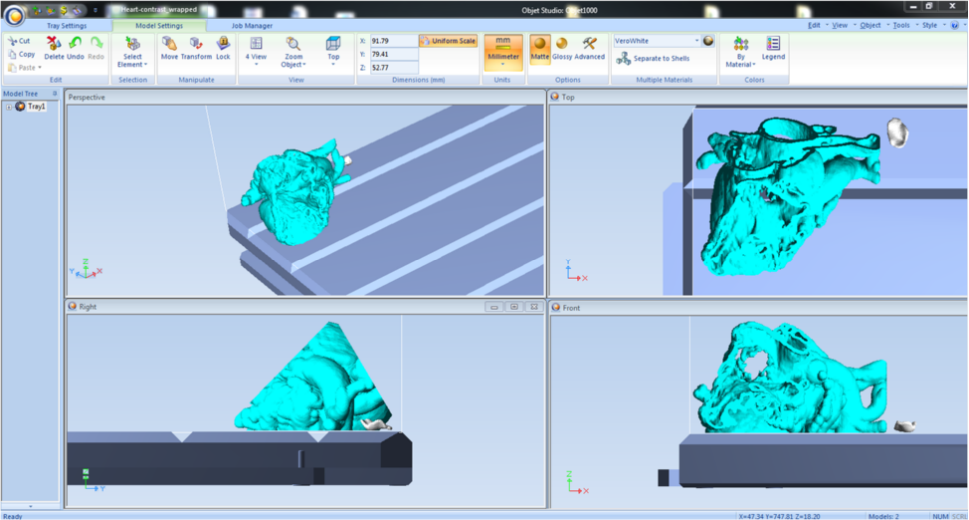
4 window zoomed Northeastern view of the models on the build tray

**Fig. 36:**
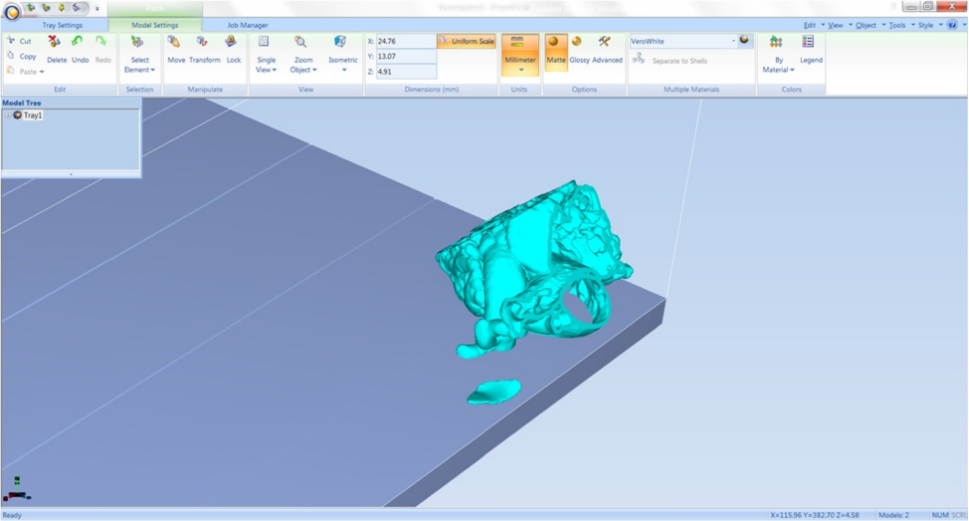
Single zoomed Northeastern view of the models on the build tray

**Fig. 37:**
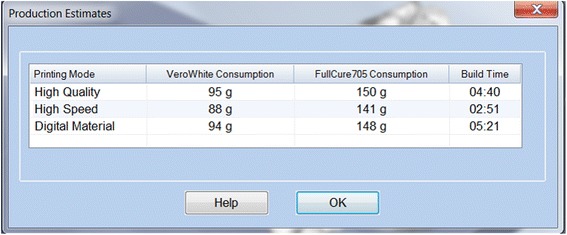
Production estimate window

**Fig. 38:**
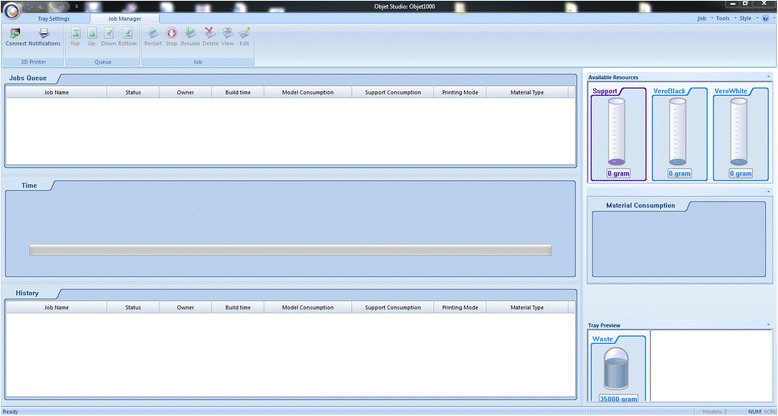
Job manager module

1. Start ***Objet Studio*** by double-clicking on the Objet Studio icon  on your Desktop. A dialog box will open, asking you to select a printing server connection. We will not have a server at RSNA, so we will select “No” (Fig. 29). This will allow us to work off-line. The main screen of ***Objet Studio*** will load as shown in Fig. 30.
2. To import the trimmed heart and the VSD patch STL file, click the upper left menu icon **Insert** , and select both STL files from the dialog box that opens by left mouse clicking (Fig. 31). (*Hint: make sure that you press and hold*  *on your keyboard before left clicking on the STL files)*. Our models will be automatically imported onto the software representation of the build tray of the printer (Fig. 32). The orientation of the heart and VSD is optimized so that the support material needed for the 3D printing job can be minimized. For example, it would be possible, but far less efficient, to print the patch standing “upright” rather than “lying down”.
3. Visualizing the model on the build tray. The default view of the entire build tray of the 3D printer is in a single isometric view. In the tab **Model Settings** you can zoom into the models in this view by clicking the icon **Zoom Tray**, and selecting **Zoom Selected Object**. The result will appear as shown in Fig. 33.
  In practice, with several objects on the build tray, it is important to be able to change the orientation of the model. This can be done by choosing **Isometric View**, and navigating the pull-down menu to **Top View**, then repeating the zoom by clicking the icon **Zoom Tray** and selecting **Zoom Selected Object**. This series of commands will move the model on the tray with the result as shown in Fig. 34. For certain printing jobs it is advantageous to study the model in a 4-axis view. To do this, click the icon **Single View** and select **4 View**. To make your screen appear as in Fig. 35, click on each individual window, make sure that the heart and the Patch models are selected by single-clicking on them, and select **Zoom Object** for a close-up view. To complete further operations on this model, return to the single window view by clicking the **4 View** icon and then selecting **Single View**. Then click on **Isometric View**, and select the **Northeast Orientation** from the pull-down menu. Zoom into the selected model by clicking **Zoom Object**. This would appear as shown in Fig. 36.
4. Next, we will assign the material of choice in order to build the model and start the 3D printing job. Click on the tab labeled **Tray Settings**. We will change the material settings to **Tango+**, an elastomeric material. To do this, select the first material pull-down menu on the right of the **Tray Setting** menu and select **Tango+**. (*Hint: Single Material should be checked and Multiple Materials unselected)*. Our model will change color, reflecting the printing in **Tango+**. There will be a confirmatory dialog box pop-up.
5. Estimating the build time and material usage before sending to the 3D Printer. Simply click the **Estimate** icon  and ***Objet Studio*** will prepare a material usage estimate and build time. This icon can be found in the upper left of our screen and selecting it will open up the **Production Estimate** window (Fig. 37). Due to the lack of a server, ***Objet Studio*** will not be actively connected to a printer. Build times and consumption of material can vary depending on the printer. You can now close the **Production Estimate** window by selecting **OK**.
6. In a production environment where the printer is connected to a print server, we would then proceed to the **Job Manager** tab to connect to the printer, send the job, and ensure that the material store in the printer is sufficient (Fig. 38).

## Conclusions

While medical 3D printing expands rapidly to becoming part of routine clinical practice in hospitals throughout the world, the expertise of generating and tailoring STL files remains in a small niche that traditionally has been centered in an engineering milieu. The hands-on material presented and illustrated will expand the expertise, and ownership, of image post-processing required for 3D printing among the radiology community. As precision medicine and patient-centric care is realized via custom-designed 3D printed models, knowledge and technical aptitude for STL files will be for the many, rather than just for the few.

## Abbreviations

3D: three dimensional
AF: additive fabrication
AM: additive manufacturing
CAD: computer-aided design
CHD: congenital heart disease
CT: computed tomography
DICOM: digital imaging and communications in medicine
DORV: double outlet right ventricle
HU: hounsfield units
MRI: magnetic resonance imaging
RF: rapid fabrication
RP: rapid prototyping
RSNA: Radiological Society of North America
STL: standard tessellation language
VSD: ventricular septal defect
